# ST2 blockade modulates IL-33–driven immune responses and is associated with reduced hepatic fibrosis in alveolar echinococcosis

**DOI:** 10.1186/s13071-026-07462-6

**Published:** 2026-06-16

**Authors:** Emad Shamsan, Liu Chuanchuan, Fan Haining

**Affiliations:** 1 College of Clinical Medicine, Qinghai University, Xining, 810001 China; 2 Affiliated Hospital of Qinghai University, Xining, 810001 China; 3 College of Medical Sciences, Taiz University, Taiz, Yemen

**Keywords:** *E. multilocularis*, Liver fibrosis, Anti-ST2 therapy, IL-33/ST2 axis, Immune modulation, Alveolar echinococcosis

## Abstract

**Background:**

Infection with *Echinococcus multilocularis* causes alveolar echinococcosis (AE), a severe parasitic disease characterised by progressive hepatic infiltration and fibrosis. Current treatment options remain limited, and the immune-mediated mechanisms underlying fibrosis are not fully understood. This study investigated the role of the IL-33/ST2 axis in AE-associated immunopathology and evaluated the effects of ST2 blockade in an experimental model.

**Methods:**

A murine model of AE was established in C57BL/6 mice, which were allocated to untreated infected, anti-ST2–treated infected and uninfected control groups. Liver pathology and fibrosis were assessed using haematoxylin and eosin staining, Masson’s trichrome staining and transmission electron microscopy. Immune and fibrotic markers were analysed by immunohistochemistry and immunofluorescence. In parallel, liver samples from patients with AE were examined to evaluate translational relevance. Systemic cytokine profiles were quantified to assess immune modulation.

**Results:**

Analysis of human AE liver samples showed an increased expression of IL-33/ST2 and associated immune and fibrotic markers, consistent with findings in the experimental mouse model. In infected mice, ST2 blockade significantly reduced metacestode lesion size and hepatic tissue invasion. This was accompanied by reduced fibrosis, as indicated by lower expression of α-smooth muscle actin, collagen I, matrix metalloproteinase-9 and tissue inhibitor of metalloproteinases-1. Anti-ST2 treatment was also associated with reduced hepatic infiltration of CD4⁺ T cells, B cells and M2 macrophages, along with decreased systemic levels of IFN-γ, TNF-α, IL-4, IL-13, IL-17 and IL-1β.

**Conclusions:**

ST2 blockade reduced metacestode growth and was associated with attenuated hepatic inflammation and fibrosis, supporting a role for IL-33/ST2 signalling in AE progression. Given the concurrent reduction in parasite burden, further studies are needed to determine whether the anti-fibrotic effects of ST2 inhibition are independent of parasite control.

**Graphical Abstract:**

**Figure:**
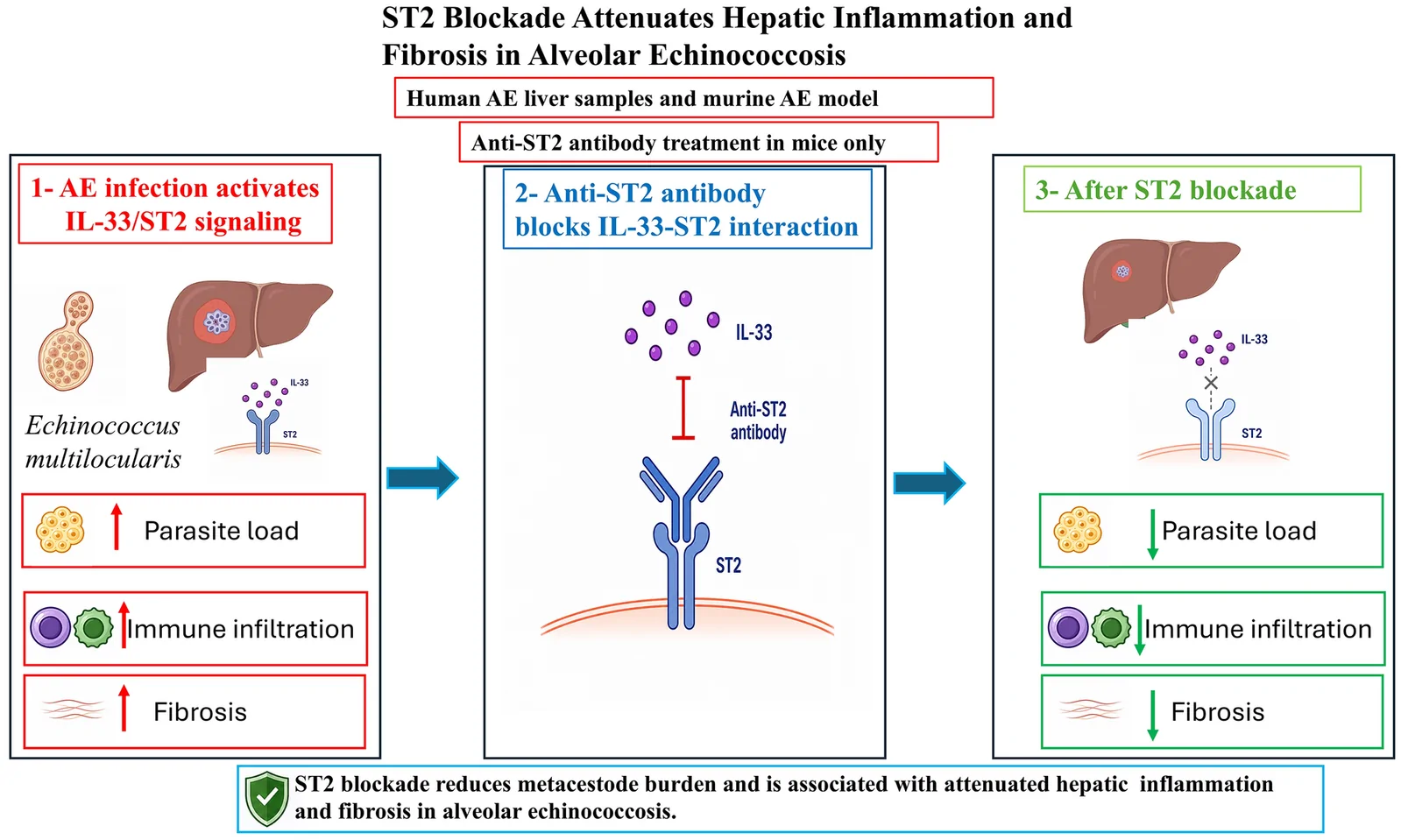

**Supplementary Information:**

The online version contains supplementary material available at 10.1186/s13071-026-07462-6.

## Background

*Echinococcus multilocularis*, the causative agent of alveolar echinococcosis (AE), is a parasitic cestode that primarily infects the liver, leading to progressive tissue infiltration, fibrosis and eventual organ dysfunction [1]. Clinically, AE behaves similarly to a malignant tumour owing to its invasive growth pattern and its capacity to destroy hepatic tissue. Current therapeutic strategies, including surgical resection and long-term antiparasitic chemotherapy, are often insufficient to fully control disease progression or reverse established fibrosis. This highlights the need for novel therapeutic approaches that target host pathological responses in addition to parasite elimination [2]. Fibrosis in chronic liver diseases is largely regulated by immune-mediated processes that control extracellular matrix (ECM) deposition and tissue remodelling [3]. Among the signalling pathways involved in immune regulation and tissue repair, the interleukin-33 (IL-33)/ST2 axis has emerged as a key mediator of inflammatory and fibrotic responses [4, 5]. IL-33, a member of the IL-1 cytokine family, is released as an alarmin following tissue damage and exerts its biological effects by binding to the membrane-bound receptor ST2L, thereby activating type 2 inflammatory responses and contributing to tissue repair in multiple disease contexts, including pulmonary inflammation, asthma and sepsis [6]. The activation of this signalling pathway promotes type 2 immune responses, immune cell recruitment and fibroblast activation, while soluble ST2 (sST2) acts as a decoy receptor that limits IL-33 signalling [7]. Although IL-33/ST2 signalling has been implicated in various diseases, its context-dependent roles in regulating immune cell subsets, including Th1, Th17 and regulatory cells, remain incompletely understood. Further research is therefore needed to clarify its mechanistic and therapeutic potential [8, 9].

Recent studies have shown that blocking ST2 signalling can reduce fibrosis by altering immune cell activation and cytokine production. Although the role of ST2 during *E. multilocularis* infection has been previously investigated, its specific contribution to fibrogenesis and the therapeutic potential of ST2 inhibition remain poorly defined [10]. In the context of AE, recent studies have shown that endogenous IL-33 is highly expressed within periparasitic tissues and plays an important role in disease progression. Experimental infection studies have demonstrated that IL-33 deficiency results in smaller metacestode lesions, indicating that IL-33 can promote parasite growth during late-stage infection [11]. Mechanistically, IL-33 contributes to the establishment of a tolerogenic microenvironment by driving Th2-associated cytokine responses, promoting M2-like macrophage polarisation and supporting regulatory immune networks that facilitate parasite persistence [11]. Beyond parasitic diseases, the IL-33/ST2 axis has also been implicated in other fibrotic disorders, where IL-33 signalling promotes fibroblast activation, collagen production and tissue remodelling [12].

Despite these advances, the specific role of ST2 signalling in the development of hepatic fibrosis during *E. multilocularis* infection remains incompletely understood. In particular, the spatial expression patterns of IL-33/ST2 signalling components in human AE lesions and their relationship with fibrotic remodelling have not been comprehensively characterised. Furthermore, the therapeutic potential of ST2 blockade on parasite-associated fibrosis has not been fully explored in experimental AE.

In the present study, we investigated the role of IL-33/ST2 signalling during AE progression using a C57BL/6 mouse model and compared these findings with pathological features observed in liver tissues from patients with AE. ST2 blockade was performed only in the experimental mouse model, whereas human liver samples were analysed to evaluate the translational relevance of the observed immunopathological patterns. Using a multimodal approach, including histopathology (haematoxylin and eosin (H&E) and Masson’s trichrome staining), immunohistochemistry (IHC) for fibrotic markers (α-smooth muscle actin (α-SMA), collagen I, matrix metalloproteinase-9 (MMP-9) and tissue inhibitor of metalloproteinases-1 (TIMP-1)) and ultrastructural evaluation by transmission electron microscopy (TEM), we characterised lesion development and ECM remodelling. Parallel immune profiling using enzyme-linked immunosorbent assay (ELISA) and immunofluorescence (IF) was performed to evaluate changes in key immune cell populations, including CD4⁺ T cells, M2 macrophages, NK cells and B cells. By integrating findings from experimental and human tissues, this study aimed to clarify the contribution of IL-33/ST2 signalling to immune regulation and fibrotic pathology during AE.

## Methods

### Human AE liver samples

Formalin-fixed paraffin-embedded (FFPE) liver tissue blocks were obtained from five patients with confirmed AE who underwent surgical resection. Control liver tissues (*n* = 5) were collected from histologically normal liver margins obtained during resections performed for benign tumours or metastatic disease unrelated to infectious or fibrotic conditions. All samples were deidentified. Details regarding ethics approval and informed consent are provided in the ethics statement.

### Animals

The 8- to 10-week-old C57BL/6 mice were used in this study, a strain commonly employed in immunological research because of its well-characterised immune responses [13]. The mice were obtained from Shanghai SLAC Laboratory Animal Co., Ltd., a certified supplier of specific pathogen-free (SPF) animals. All animals were housed under SPF conditions at the Animal Laboratory Center of Shanghai University of Traditional Chinese Medicine. Mice were maintained in a controlled environment on a 12 h light/dark cycle with ad libitum access to autoclaved food and water, in accordance with standard laboratory practices [14].

### Echinococcus multilocularis infection

Metacestode tissue of *E. multilocularis* was aseptically harvested from the peritoneal cavities of chronically infected BALB/c mice [15]. C57BL/6 mice were used for experimental infection because of their well-characterised immune responses and the availability of immunological reagents, making them suitable for immunological analyses [16]. Parasites derived from BALB/c mice are compatible with C57BL/6 mice, and this approach is commonly used in experimental models of AE. Metacestode tissue was homogenised and filtered through a sterile 50-μm nylon mesh to obtain a suspension containing approximately 100 freshly prepared acephalic vesicular cysts. The vesicles were suspended in 100 μL of sterile normal saline (NS) and intraperitoneally injected into each mouse. Control mice received 100 μL of NS alone. The infection protocol was adapted from previously described methods, except that the vesicles were suspended in NS rather than Roswell Park Memorial Institute (RPMI)-1640 medium. Mice were monitored for disease progression following infection. In this model, early parasite establishment and hepatic lesions were detectable by ultrasound at approximately 3 weeks post infection. On the basis of this observation, treatment was initiated at week 3 to evaluate the effects of ST2 blockade during the early phase of infection.

### Experimental groups

Mice were randomly assigned to three experimental groups (*n* = 7 per group unless otherwise stated). The infected group included mice infected with *E. multilocularis* without treatment. The infected + anti-ST2 group included infected mice treated with 50 µg of anti-ST2 neutralising antibody. The control group included uninfected mice receiving phosphate-buffered saline (PBS) only.

### Anti-ST2 treatment

**Fig. 1:**
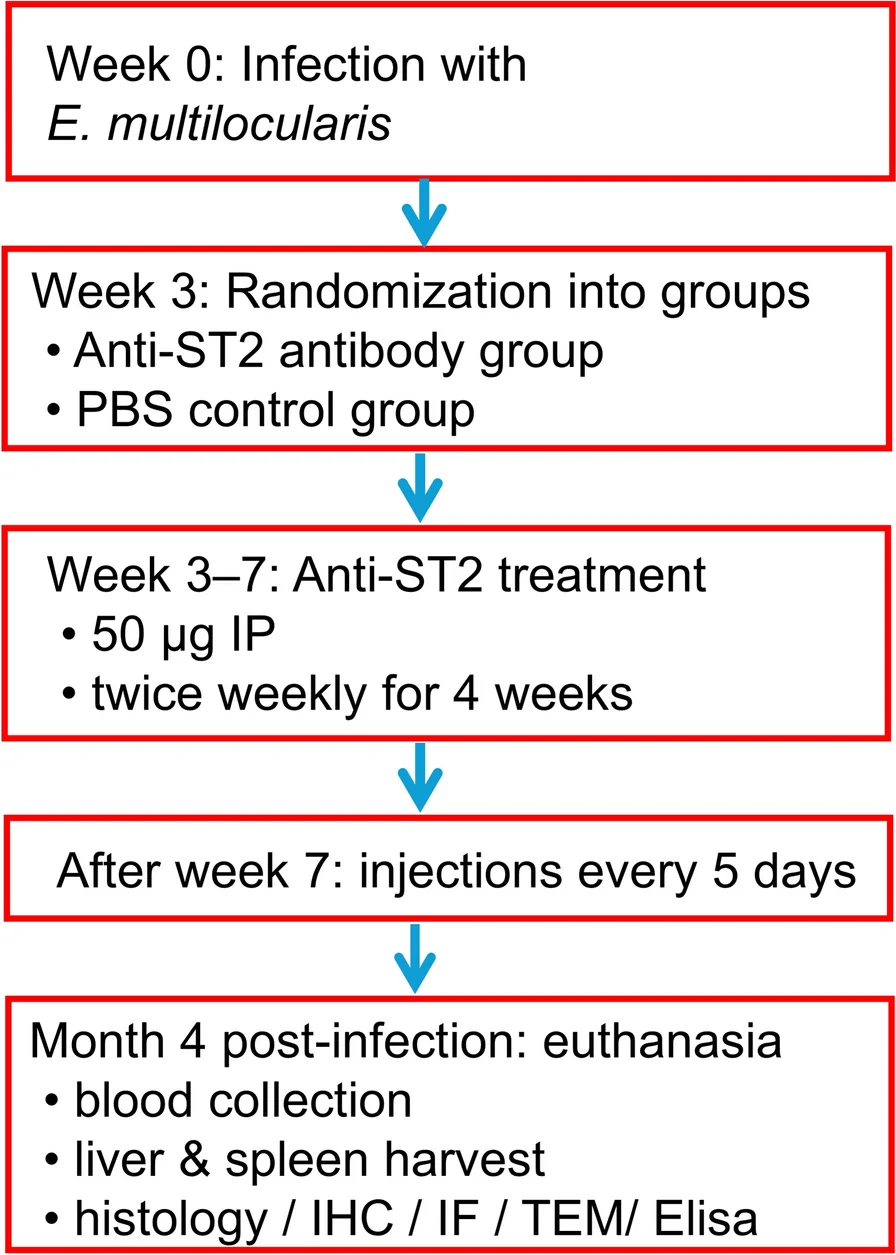
Schematic timeline of the experimental design. C57BL/6 mice were infected with *Echinococcus multilocularis*, treated with an anti-ST2 neutralising antibody from week 3 post infection and sampled at 4 months post infection

A monoclonal anti-mouse ST2 neutralising antibody (anti-IL1RL1/ST2/IL-33R; human IgG2σA isotype) produced in CHO cells was used for in vivo blockade. Mice received 50 µg per injection (~2.5 mg/kg for a 20 g mouse) in a total volume of 100 µL PBS intraperitoneally. Treatment was initiated 3 weeks post infection. Injections were administered twice weekly for 4 weeks, followed by administration every 5 days thereafter until the end of the study. Control mice received 100 µL PBS according to the same schedule. The treatment regimen was adapted from previous studies demonstrating the immunomodulatory efficacy of anti-ST2 in parasitic disease models [17]. The experimental timeline is shown in Fig. 1.

### Sample collection

At 4 months post infection (week 16; see Fig. 1), mice were euthanised. Blood samples were collected via cardiac puncture, and serum was isolated and stored at −80 °C for cytokine analysis. Liver and spleen tissues were harvested and processed for downstream analyses, including histopathology (fixed in 10% formalin), immunohistochemistry (IHC; paraffin embedding), immunofluorescence (IF; OCT embedding) and ultrastructural analysis (TEM; glutaraldehyde fixation).

### Histological analysis

Liver and spleen tissues were fixed, processed and embedded in paraffin following standard histological procedures. Paraffin-embedded sections (4 µm) were cut using a microtome. Haematoxylin and eosin (H&E) staining was performed to assess general tissue morphology according to established protocols [18], whereas Masson’s trichrome staining was used to evaluate collagen deposition and hepatic fibrosis.

Stained slides were examined using a light microscope (Olympus MX63L), and digital images were acquired using a Pannoramic 250 digital slide scanner (3DHISTECH, Hungary). Slides were examined at low magnification to assess overall tissue architecture. For quantitative analysis, three representative fields per section were captured at 200× magnification. Three sections per tissue from each mouse and three mice per group were analysed. Fibrotic areas were delineated and quantified using Image-Pro Plus 6.0 (Media Cybernetics, USA), and results were expressed as the percentage of fibrotic area relative to the total field area.

### Immunohistochemistry and immunofluorescence

For both human and murine samples, consecutive sections were prepared from formalin-fixed, paraffin-embedded (FFPE) tissue blocks and processed using standardised protocols to allow comparative analysis. IHC was performed using primary antibodies from Proteintech Group, Inc. (Rosemont, IL, USA), including collagen I, α-SMA, MMP-9, TIMP-1, IL-33, ST2 and IL-6. Antibody binding was visualised using a DAB chromogenic detection system according to the manufacturer’s instructions.

IF staining was performed to identify immune cell subsets using primary antibodies from MedChemExpress (MCE), including CD4, CD206 (M2 macrophage marker), NK1.1 (NK cell marker) and CD19 (B cell marker). Sections were incubated with species-appropriate fluorophore-conjugated secondary antibodies and counterstained before imaging.

Stained slides were examined using both a light microscope (Olympus MX63L) and a fluorescence microscope. Quantitative analysis of staining intensity and positive cell area was performed on five randomly selected perilesional fields per sample using ImageJ software [19, 20].

### Transmission electron microscopy

Liver tissues were processed for TEM following a previously reported protocol with minor modifications [21]. Briefly, samples were fixed in 2.5% glutaraldehyde and subsequently postfixed in 1% osmium tetroxide. The specimens were dehydrated through a graded ethanol series and embedded in epoxy resin. Ultrathin sections (60–80 nm) were prepared using a Leica UC7 ultramicrotome and stained with uranyl acetate and lead citrate. The sections were then examined and imaged using a Hitachi HT7700 transmission electron microscope.

### Enzyme-linked immunosorbent assay

At the experimental endpoint, blood samples were collected. Serum was isolated by centrifugation at 3,000 rpm for 15 min at 4 °C and stored at −80 °C until analysis. Cytokine levels (IL-4, IL-13, IL-17, IFN-γ, TNF-α and IL-1β) were quantified using ELISA kits (Proteintech Group, Inc., Rosemont, IL, USA) according to the manufacturer’s instructions. Absorbance was measured at 450 nm using a Bio-Rad iMark™ microplate reader, and cytokine concentrations were calculated from standard curves. All assays were performed in duplicate.

### Statistical analysis

All data are expressed as the mean ± standard deviation (SD). Statistical analyses were performed using GraphPad Prism version 9.0 (GraphPad Software, USA). Differences among multiple groups were assessed using one-way analysis of variance (ANOVA), followed by Tukey’s post hoc test for multiple comparisons. Differences between two groups were assessed using an unpaired Student’s *t*-test. A *P*-value < 0.05 was considered statistically significant.

## Results

To investigate the role of IL-33/ST2 signalling in AE, we assessed activation of the IL-33/ST2 axis in liver tissues from patients with AE and compared these findings with those observed in the experimental murine model. Therapeutic ST2 blockade was performed exclusively in infected C57BL/6 mice, and its effects were evaluated using histological, immunological and imaging analyses. This approach allowed integration of human pathological signatures with experimental outcomes.

### Activation of the IL-33/ST2 axis in human and experimental AE

**Fig. 2:**
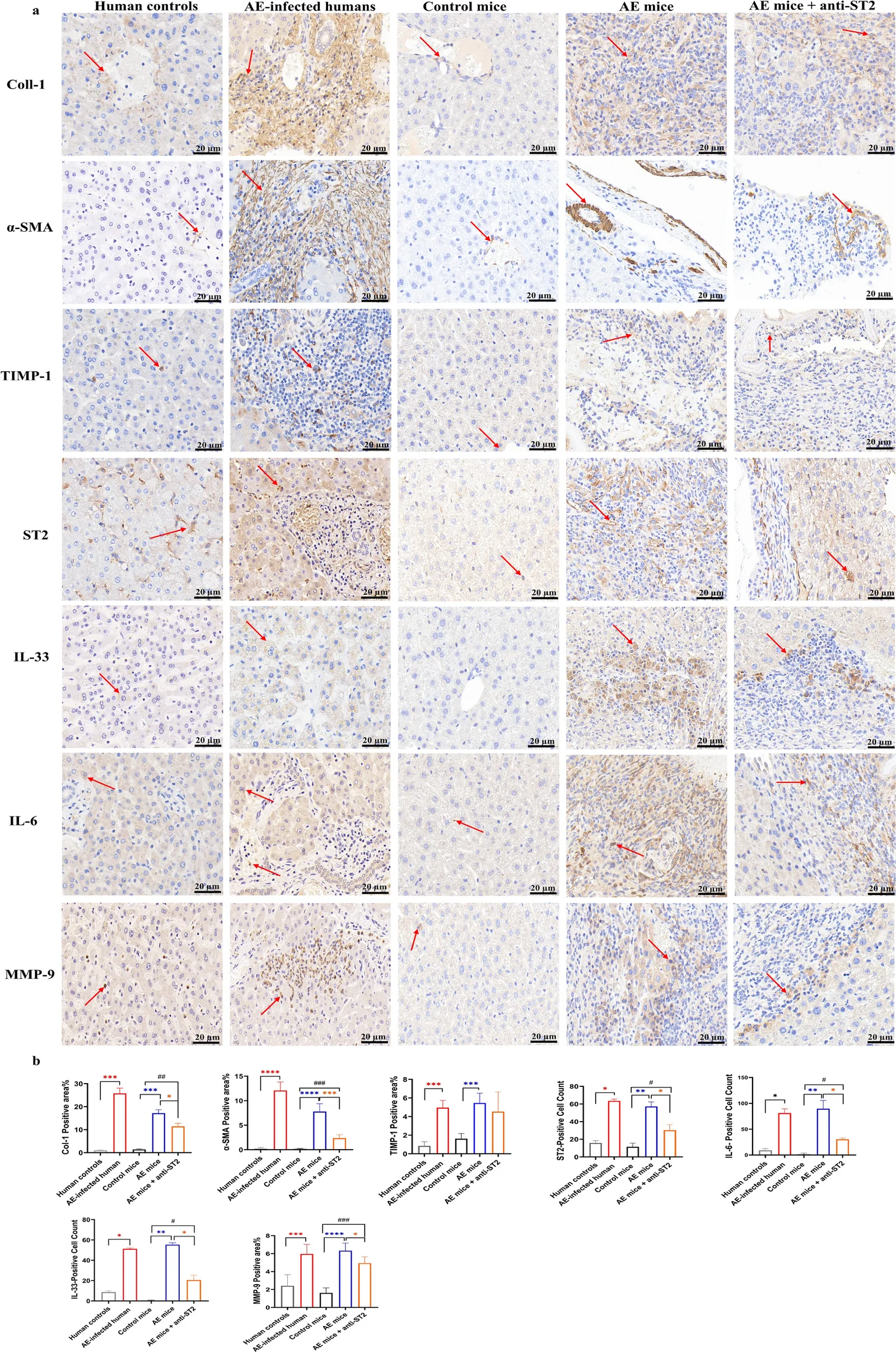
Activation of the IL-33/ST2 pathway in human AE and its reduction following anti-ST2 treatment in mice. **a**, Representative IHC images of collagen I, α-SMA, TIMP-1, ST2, IL-33, IL-6 and MMP-9 in liver sections from controls, patients with AE and mice (control, infected and anti-ST2-treated). **b**, Quantification of IHC staining intensity. Scale bar: 20 µm; magnification, 400×. *P < 0.05, **P < 0.01, ***P < 0.001 versus the AE-infected group; #*P* < 0.05, ##*P* < 0.01 versus the control group

The IHC analysis of liver specimens from patients with AE showed marked upregulation of ST2, IL-33 and the fibrotic markers α-SMA and collagen I within parasitic lesions compared with non-AE control tissues (Fig. 2). A similar pattern of IL-33/ST2 axis activation and fibrotic marker expression was observed in the murine AE model, indicating conservation of this signalling axis between human and experimental disease.

In infected mice, administration of anti-ST2 antibodies significantly reduced the expression of ST2 and IL-33 within hepatic lesions. This reduction was accompanied by decreased levels of α-SMA, collagen I and IL-6 compared with untreated infected controls. These findings indicate that modulation of the IL-33/ST2 axis in vivo is associated with reduced fibrotic marker expression in experimental AE.

Overall, these results support a role for IL-33/ST2 signalling in AE-associated immunopathology and provide a rationale for therapeutic targeting in the murine model.

### Immune cell infiltration in human and experimental AE

**Fig. 3:**
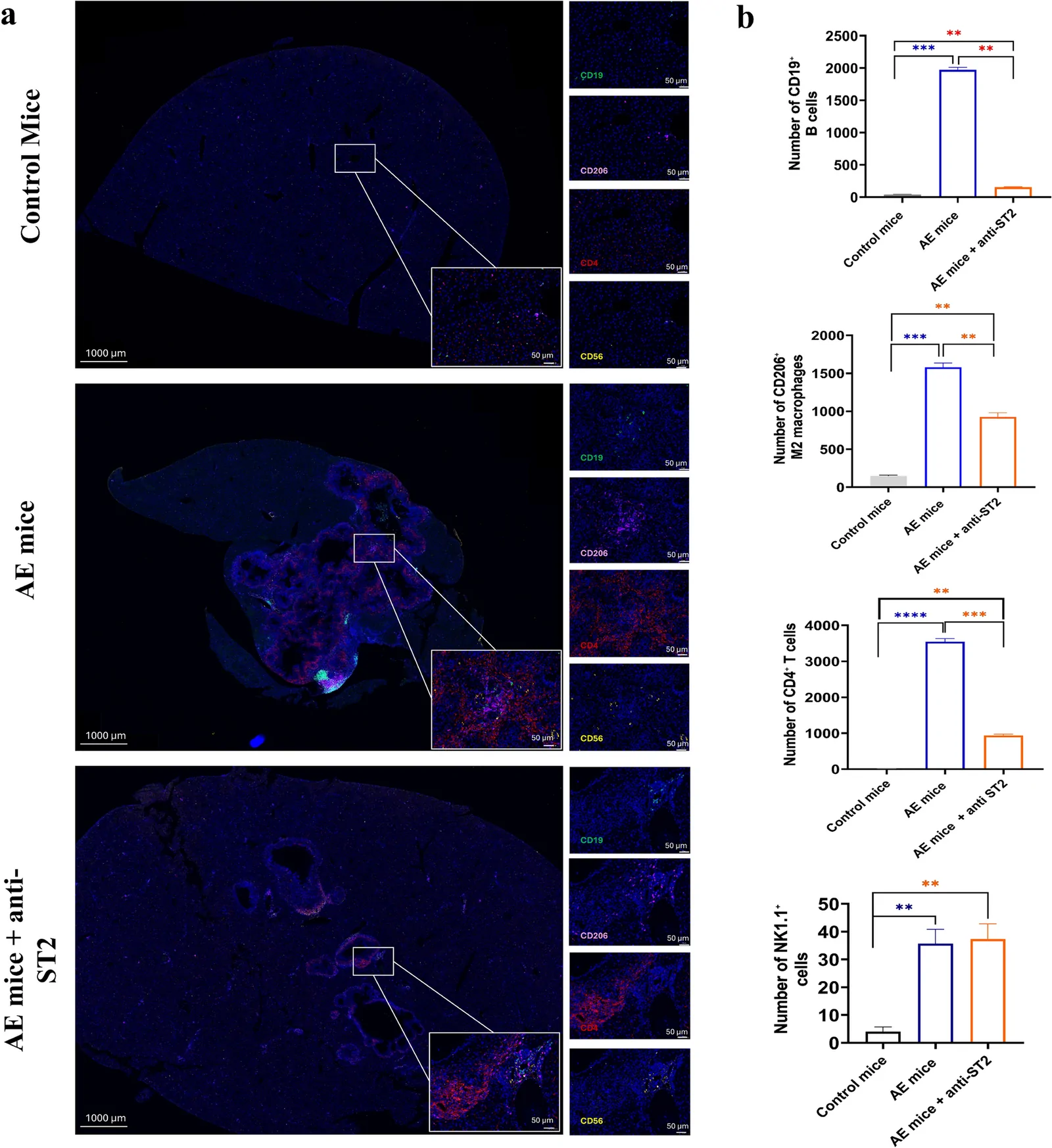

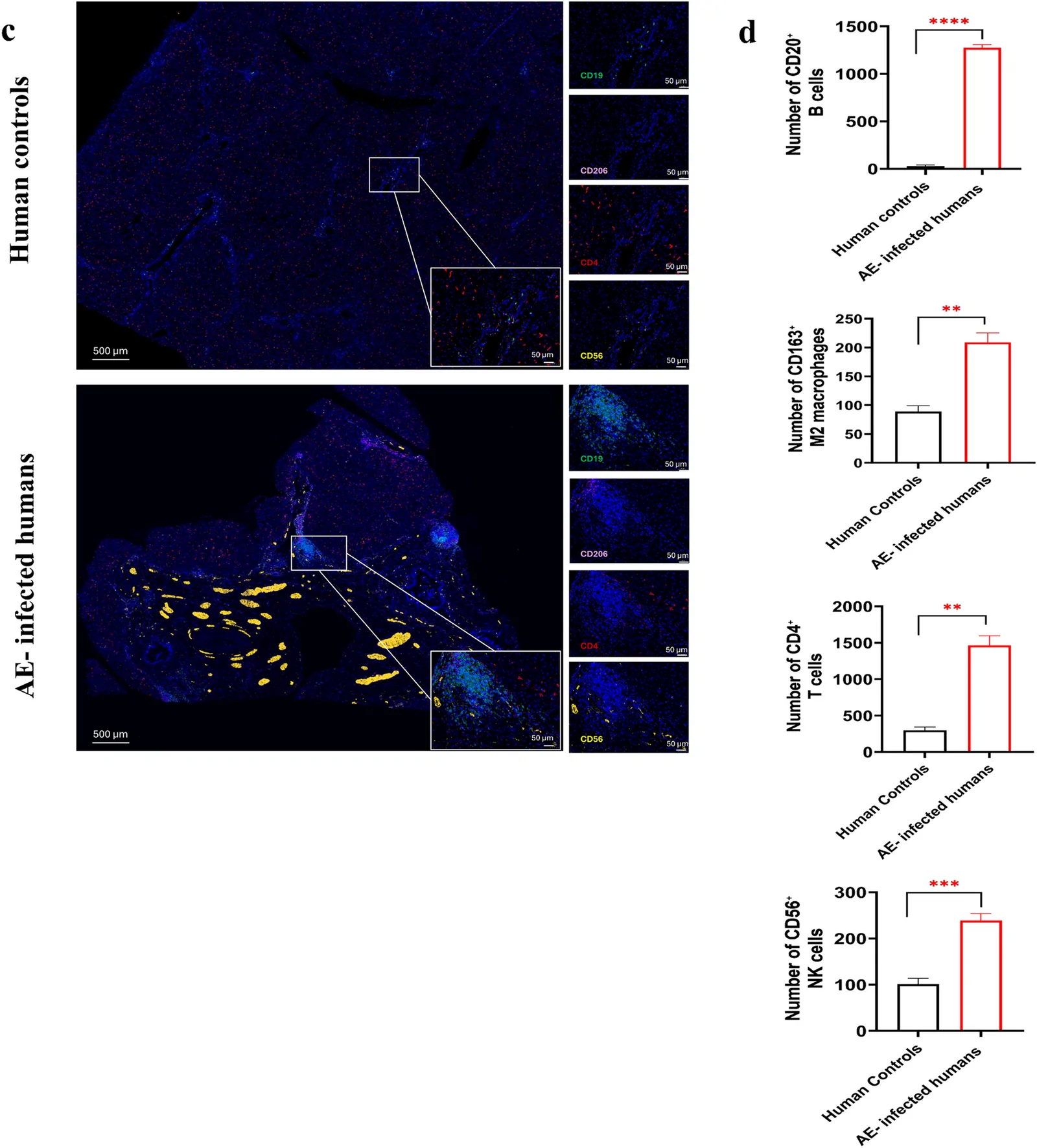
Conserved immune cell infiltration in murine and human AE lesions and modulation by ST2 blockade. **a**, **b**, IF images and quantification of CD4⁺ T cells, B cells, M2 macrophages and NK cells in control, AE-infected and anti-ST2-treated mouse livers. **c**, **d**, IF images and quantification of the same cell types in human AE and control liver tissues. Images are shown at high (20×; scale bar, 50 µm), low (2×; scale bar, 500 µm) and 1.2× (scale bar, 1,000 µm) magnification. *P < 0.05, **P < 0.01, ***P < 0.001. *n* = 3–4 per group

Immunophenotypic analysis revealed a pronounced accumulation of immune cell populations within AE lesions in both human and murine tissues (Fig. 3). In mouse liver tissues, representative immunostaining images (Fig. 3a) showed increased infiltration of CD4⁺ T cells, B cells, M2 macrophages and NK cells in AE-infected mice compared with controls, which was supported by quantitative analysis (Fig. 3b). Anti-ST2 treatment significantly reduced the recruitment of CD4⁺ T cells, B cells and M2 macrophages. In contrast, NK cell infiltration was increased in infected mice but was not significantly altered by ST2 blockade.

In human AE liver specimens, representative histological images (Fig. 3c) demonstrated marked infiltration of immune cells. Quantitative analysis (Fig. 3d) confirmed that all examined immune cell populations were significantly increased compared with non-AE control tissues. The concordance between murine and human immune profiles highlights a conserved pattern of immune remodelling in AE. Collectively, these findings suggest that IL-33/ST2 signalling may be associated with changes in the immune microenvironment in AE, whereas ST2 blockade is associated with reduced accumulation of selected immune cell populations in the murine model.

### ST2 blockade reduces parasite burden and systemic disease in chronic AE

**Fig. 4:**
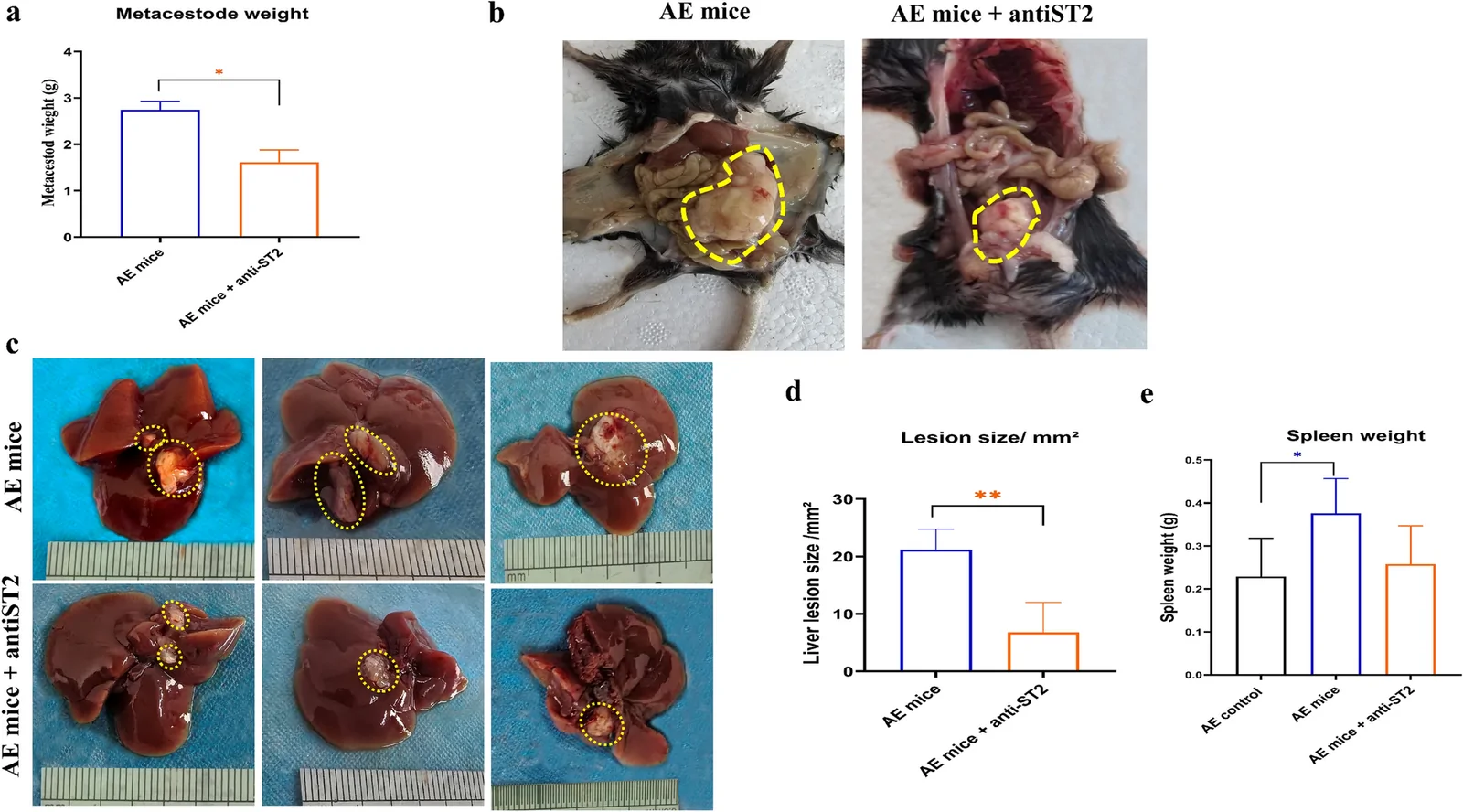
Parasite burden and systemic disease at 125 days post infection. **a**, Metacestode weight, **b**, representative abdominal images, **c**, liver lesions, **d**, quantification of liver lesion size and **e**, spleen weight. Data are presented as the mean ± SEM (*n* = 5). Statistical analysis was performed using one-way ANOVA followed by Tukey’s post hoc test; *P < 0.05, **P < 0.01

Having established that anti-ST2 treatment effectively targets the IL-33/ST2 pathway at the molecular level, we next assessed its impact during chronic infection. Mice treated with anti-ST2 showed a lower parasite burden. At 125 days post infection, untreated mice developed substantial metacestode masses (2.29 ± 0.45 g), whereas anti-ST2 treatment was associated with a 39% reduction in metacestode weight (1.40 ± 0.26 g; *P* < 0.05; Fig. 4a, b), suggesting a possible association between IL-33/ST2 pathway modulation and parasite persistence.

Consistent with reduced parasite burden, hepatic lesions appeared smaller in treated mice than in infected controls (*P* < 0.01; Fig. 4d), with several treated animals showing near-complete resolution of detectable lesions (Fig. 4c). Liver weight, which was slightly elevated in infected mice, showed a modest reduction following treatment.

Systemically, AE infection induced significant splenomegaly, a hallmark of chronic immune activation. Spleen weight increased from 0.18 ± 0.06 g in controls to 0.42 ± 0.13 g in infected mice (*P* < 0.01). Anti-ST2 treatment was associated with a partial reduction in splenomegaly (0.34 ± 0.12 g; *P* < 0.05; Fig. 4e), consistent with changes in systemic immune responses. Representative images illustrating spleen size reduction are provided in Additional file 2: Supplementary Fig. S4.

### ST2 blockade is associated with reduced hepatic fibrosis and improved liver architecture

**Fig. 5:**
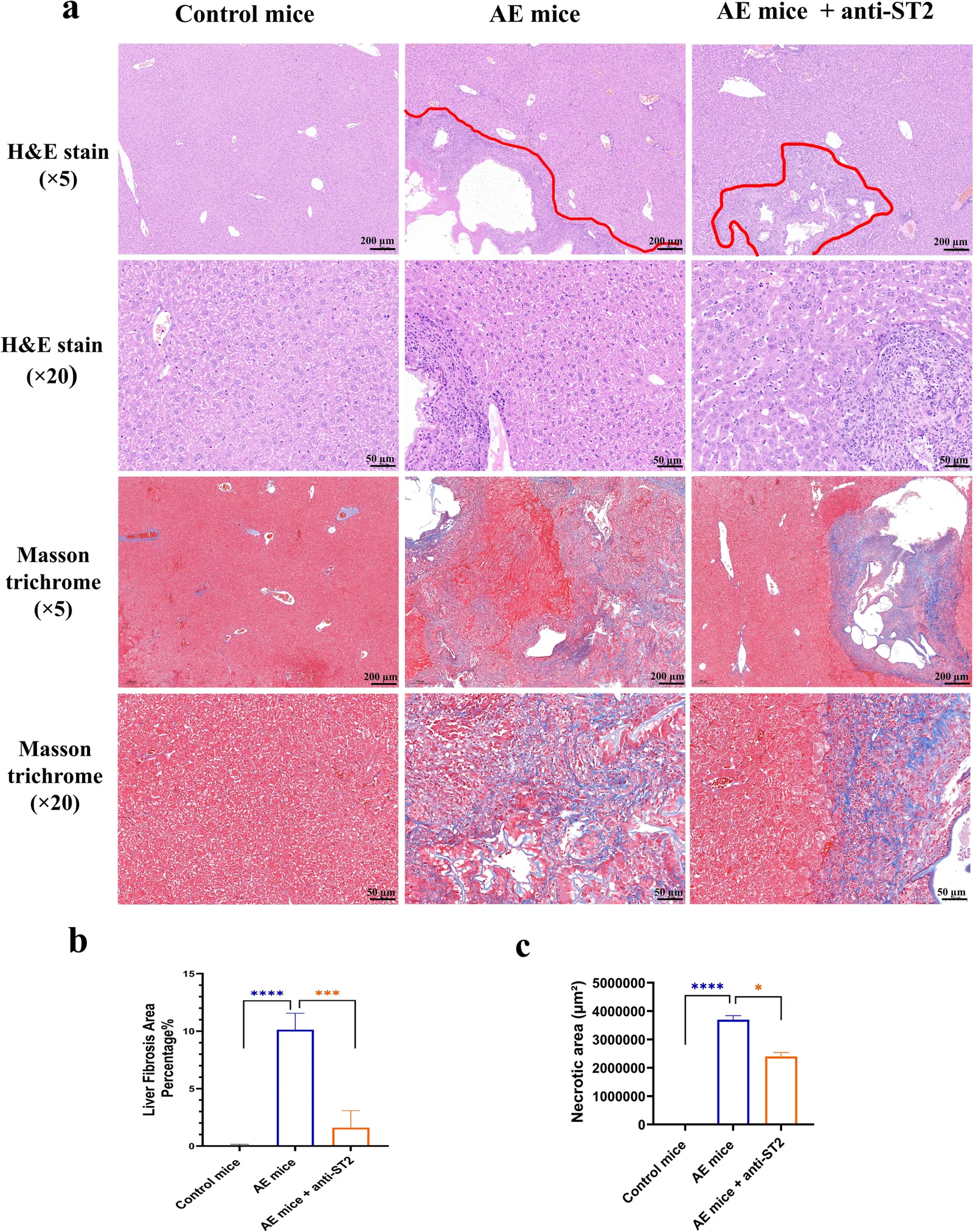
ST2 blockade attenuates hepatic fibrosis and improves liver architecture in chronic AE. **a**, Representative H&E and Masson’s trichrome staining of liver tissues from AE-infected and anti-ST2-treated mice at low (5×) and high (20×) magnification. **b**, Quantification of hepatic fibrotic area (% of total liver area). **c**, Quantification of necrotic area (µm^2^). Dashed red lines indicate lesion boundaries. Data are presented as the mean ± SEM (*n* = 3 per group). Statistical analysis was performed using one-way ANOVA followed by Tukey’s post hoc test. *P < 0.05, ***P < 0.001, ****P < 0.0001

Histological examination showed that anti-ST2 treatment was associated with reduced AE-induced liver damage and fibrotic remodelling. Livers from infected mice exhibited severe disruption of hepatic architecture, extensive inflammatory cell infiltration and pronounced collagen accumulation surrounding metacestode lesions, as evidenced by H&E and Masson’s trichrome staining (Fig. 5a).

In contrast, anti-ST2-treated mice displayed improved liver architecture, characterised by expanded areas of viable hepatocytes, reduced inflammatory infiltration and decreased pericystic collagen deposition (Fig. 5a).

Quantitative analysis confirmed a significant reduction in fibrotic area, from approximately 12.5% in infected mice to approximately 2% following anti-ST2 treatment (*P* < 0.001; Fig. 5b). Similarly, the necrotic area was significantly reduced, decreasing from approximately 4.0 mm^2^ to approximately 2.5 mm^2^ (*P* < 0.05; Fig. 5c).

Collectively, these findings show that ST2 blockade was associated with reduced hepatic fibrosis and improved liver structural integrity during chronic AE.

### ST2 blockade is associated with improved hepatocyte ultrastructure

**Fig. 6:**
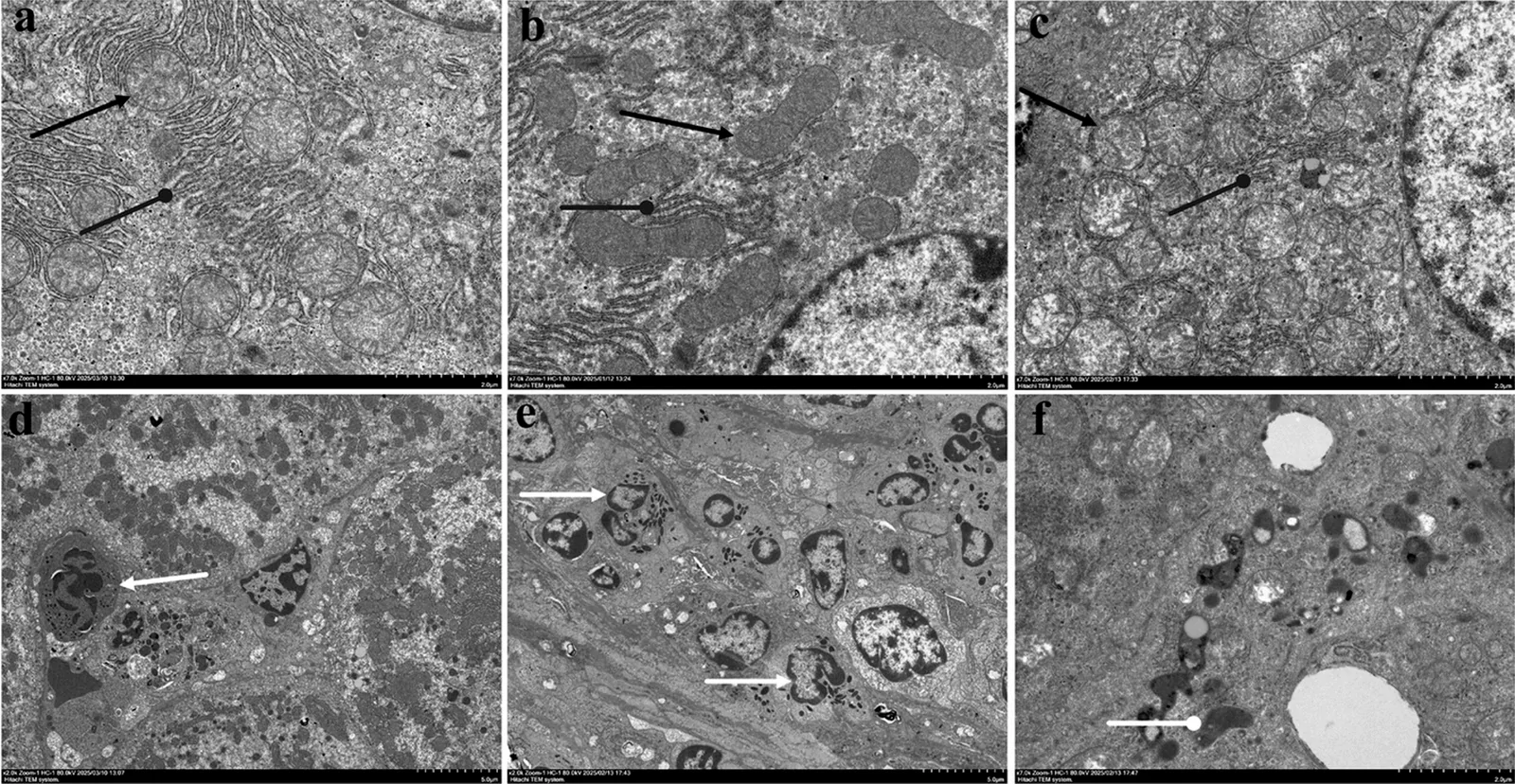
ST2 blockade is associated with improved hepatocyte ultrastructure and altered innate immune infiltration. Transmission electron microscopy (TEM) of liver tissues from control, AE-infected and anti-ST2-treated mice. **a**–**c**, Mitochondrial ultrastructure in hepatocytes. **d**–**f**, Nuclear morphology and immune cell infiltration. Black straight arrows indicate mitochondria; black circle-tipped arrows indicate cytoplasmic disorganisation; white straight arrows indicate neutrophils; white circle-tipped arrows indicate monocytes. Scale bars: 2.0 µm (**a**–**c**, 7,000×); 5.0 µm (**d**–**f**, 2,000×)

TEM revealed that anti-ST2 treatment was associated with reduced ultrastructural damage in hepatocytes. Cells from control mice exhibited normal mitochondrial morphology, with well-defined cristae, intact membranes and round nuclei containing evenly dispersed chromatin (Fig. 6a, d). In contrast, AE-infected mice showed severe cellular injury, including swollen mitochondria with disrupted cristae, cytoplasmic disorganisation and nuclei with condensed, irregular chromatin accompanied by pronounced neutrophil infiltration (Fig. 6b, e).

Hepatocytes from anti-ST2-treated mice displayed improved cellular integrity, with mitochondria showing more intact membranes and clearer cristae, along with partial restoration of nuclear morphology (Fig. 6c, f). Moreover, the immune infiltrate shifted, with monocytes predominating over neutrophils, consistent with reduced acute inflammatory infiltration and features of tissue recovery.

### ST2 blockade is associated with changes in systemic cytokine responses during AE progression

#### Early stage (60 days post infection)

Serum samples were collected at 60 days post infection to assess systemic cytokine responses. At this early stage of AE, infected mice showed increased levels of proinflammatory cytokines, with several proinflammatory cytokines significantly elevated compared with uninfected controls. However, no significant differences in serum cytokine levels were observed between infected and treated mice at this time point, indicating no substantial changes in systemic cytokine production during the early stage of infection.

#### Chronic stage (125 days post infection)

**Fig. 7:**
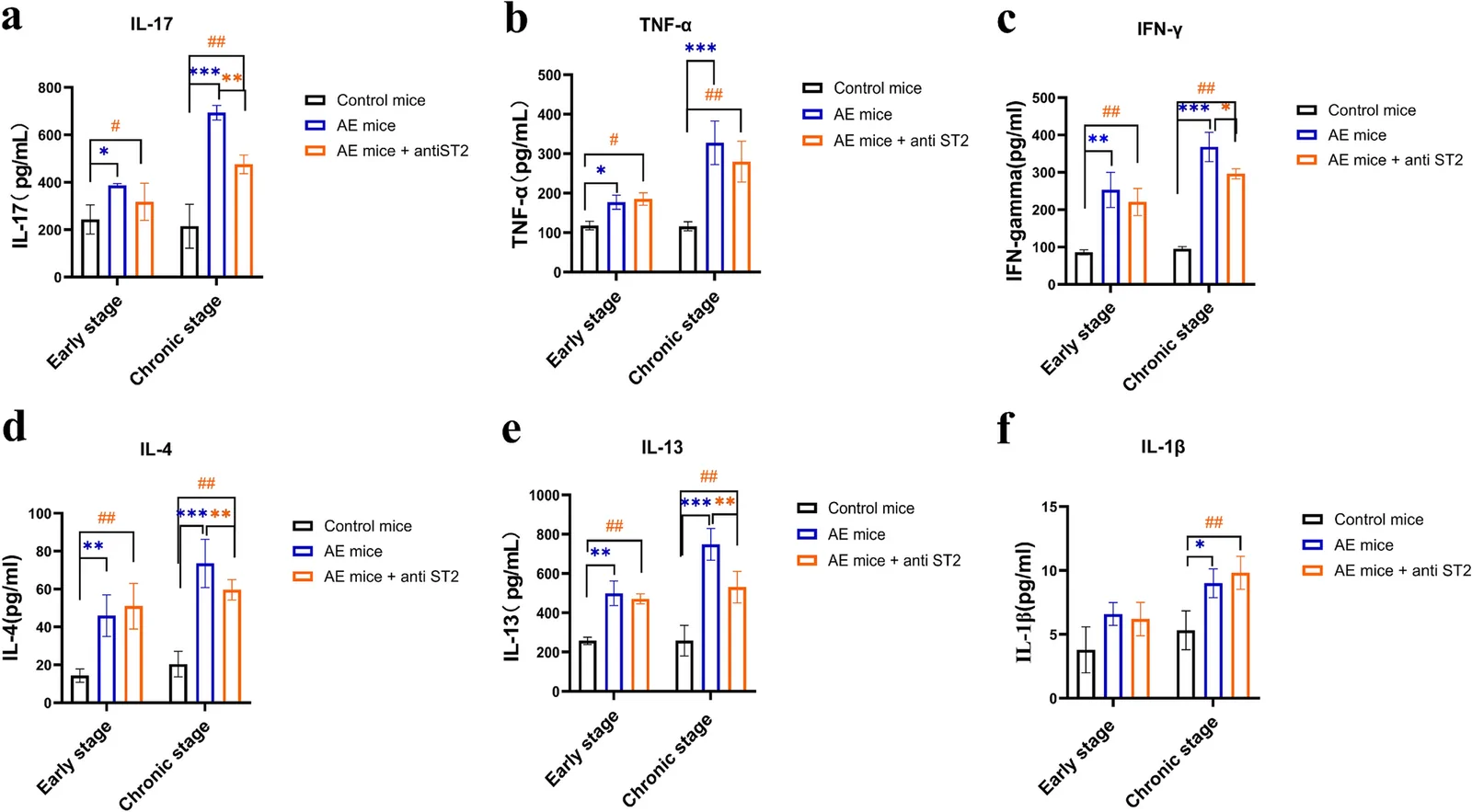
ST2 blockade modulates systemic cytokine responses in chronic AE. Serum cytokine levels were measured at 60 and 125 days post infection in control, AE-infected and anti-ST2-treated mice. **a**–**c**, Th1/Th17-associated cytokines (IFN-γ, IL-17 and TNF-α). **d**, **e**, Th2-associated cytokines (IL-4 and IL-13). **f**, Proinflammatory cytokine IL-1β. Data are presented as the mean ± SEM (*n* = 5 mice per group). Statistical analysis was performed using one-way ANOVA followed by Tukey’s post hoc test. *P < 0.05, **P < 0.01, ***P < 0.001 versus the AE-infected group; #*P* < 0.05, ##*P* < 0.01 versus the control group

At 125 days post infection, corresponding to the chronic stage of AE, ST2 blockade was associated with changes in systemic cytokine responses. Levels of the Th1/Th17-associated cytokines IFN-γ and IL-17, which were elevated in infected mice, were lower in treated mice than in infected mice (*P* < 0.05 and *P* < 0.02, respectively; Fig. 7a, c). TNF-α levels showed a downward trend but did not reach statistical significance (Fig. 7b). Chronic infection was associated with a Th2-skewed response and ST2 blockade was associated with lower levels of IL-4 and IL-13 (*P* < 0.001 and *P* < 0.03, respectively; Fig. 7d, e). In contrast, IL-1β levels remained elevated despite treatment (Fig. 7f), indicating that its production may be partially independent of IL-33/ST2 signalling. Because parasite burden was concurrently reduced by ST2 blockade, the extent to which these cytokine changes are independent of parasite burden requires further investigation. Collectively, these findings indicate an association between ST2 blockade and systemic Th1/Th17- and Th2-related cytokine changes during chronic AE.

### ST2 blockade is accompanied by changes in splenic microarchitecture and lymphocyte organisation

IF analysis of spleen sections revealed marked differences in lymphoid organisation among control, infected and anti-ST2-treated mice. In control mice, B cells and CD4⁺ T cells were abundant and displayed well-preserved splenic architecture, with B cells forming compact follicles within the white pulp and CD4⁺ T cells predominantly localised to the periarteriolar lymphoid sheaths (PALS).

**Fig. 8:**
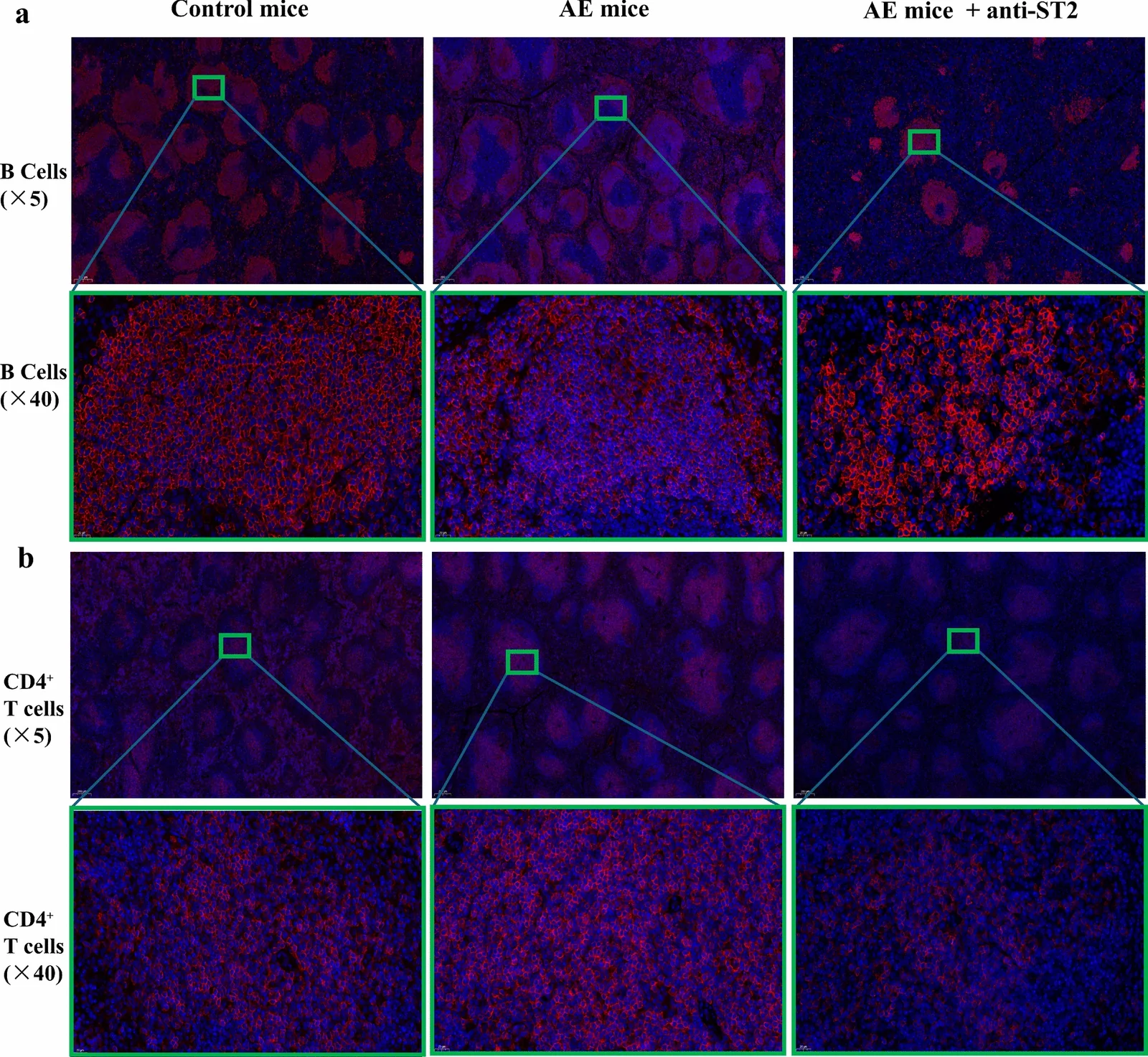
ST2 blockade alters splenic distribution of B cells and CD4⁺ T cells in *E. multilocularis*-infected mice. Representative immunofluorescence (IF) images of B cells and CD4⁺ T cells in spleen sections from control, AE-infected and anti-ST2-treated mice. Low-magnification images (5×; scale bar, 200 µm) show overall spleen architecture, whereas high-magnification images (40×; scale bar, 20 µm) show the boxed white pulp region

In contrast, infected untreated mice exhibited altered lymphocyte distribution, characterised by reduced B-cell follicular density and less well-defined organisation of CD4⁺ T cells within the white pulp, indicating disruption of normal splenic immune architecture during chronic infection. Notably, anti-ST2 treatment was associated with further alterations. B cells were markedly reduced within follicular and white pulp regions, accompanied by fragmentation of follicular structures (Fig. 8a). CD4⁺ T cells were similarly reduced in number and exhibited a dispersed, discontinuous distribution within the PALS (Fig. 8b), reflecting a pronounced loss of white pulp integrity.

These findings indicate that chronic infection disrupts splenic lymphoid organisation, while ST2 blockade is accompanied by further reductions in lymphocyte abundance and alterations in the splenic microanatomical framework that supports coordinated adaptive immune responses.

## Discussion

AE is a severe parasitic disease characterised by progressive hepatic fibrosis that mimics malignancy through its invasive growth and fibrotic remodelling [22–24]. Our study identified hyperactivation of the IL-33/ST2 pathway in liver tissues from patients with AE, highlighting its clinical relevance. In the experimental C57BL/6 murine AE model, the pharmacological blockade of ST2 was associated with reduced metacestode growth and reduced fibro-inflammatory pathology, consistent with previous reports indicating that parasite burden is a major driver of hepatic fibrosis in AE [11]. These data suggest that improvements in tissue architecture are likely secondary to reduced parasite burden rather than a direct anti-fibrotic effect of ST2 inhibition.

Histopathological analysis showed that anti-ST2 treatment was associated with decreased collagen deposition and more organised ECM structures. TEM revealed improved hepatocyte ultrastructure, including preserved mitochondrial cristae and reduced endoplasmic reticulum stress. These structural changes likely reflect reduced fibro-inflammatory injury secondary to reduced parasite burden.

Immune profiling revealed that ST2 blockade selectively reduced M2 (alternatively activated) macrophages, CD4⁺ T cells and B cells in the liver and spleen, whereas NK cells were largely unaffected. This observation is consistent with studies in viral infection models, where ST2 deficiency impairs NK cell expansion but not differentiation [25], indicating that ST2 signalling may differentially regulate distinct immune cell subsets.

At the chronic stage, serological cytokine analysis showed reduced serum levels of IL-4, IL-13, TNF-α, IFN-γ and IL-17 following anti-ST2 treatment, suggesting broad but selective immunomodulatory effects. IHC analysis further revealed decreased expression of ST2, IL-33, IL-6, α-SMA, collagen I, TIMP-1 and MMP-9 in treated mice. These observations are consistent with previous reports in murine models of parasite-associated fibrosis and chronic AE, in which IL-33/ST2 signalling promotes tissue remodelling and myofibroblast activation [10, 11, 26]. These molecular changes occurred alongside reductions in parasite burden, a primary driver of downstream fibro-inflammatory responses in AE.

IL-33/ST2 signalling is pivotal for maintaining immunosuppressive and pro-fibrotic microenvironments. In murine and human tumour microenvironment models, IL-33 promotes M2 macrophage polarisation and contributes to immunoregulatory environments [27]. Other studies in murine models have demonstrated effects on CD4⁺ T cells [28] and B cells [29]. In line with these observations, anti-ST2 treatment in our AE model reduced M2 macrophage, CD4⁺ T cell and B cell populations, consistent with a role for ST2 signalling in sustaining a pro-fibrotic and immunoregulatory niche during AE progression.

The role of IL-33/ST2 in neutrophil recruitment is supported by murine models of gout, in which this pathway drives neutrophil influx and reactive oxygen species generation [30]. Although AE is a chronic parasitic disease, the reduction in neutrophil infiltration following ST2 blockade suggests that IL-33/ST2 contributes to innate immune cell recruitment in fibrotic hepatic microenvironments.

ST2 blockade also influenced systemic immune organisation. An analysis of splenic architecture demonstrated reduced B cell and CD4⁺ T cell zones in treated animals, consistent with previous studies suggesting that IL-33/ST2 signalling modulates splenic immune responses in parasitic infection models, such as *Leishmania donovani* infection [31] and regulates immune cell function in systemic inflammatory settings, such as pregnancy models [29].

Collectively, these findings indicate that IL-33/ST2 signalling contributes to the fibro-inflammatory environment characteristic of AE through immunomodulation and support of parasite persistence. Pharmacological blockade of ST2 mitigated disease severity, reduced parasite burden and indirectly alleviated hepatic fibrosis and immune dysregulation. Importantly, no obvious systemic toxicity or organ dysfunction was observed, suggesting that targeting this pathway may have therapeutic potential. These observations are consistent with broader evidence that IL-33/ST2 shapes tumour-like microenvironments [22, 24, 32], fibrotic niches in chronic inflammatory diseases [6, 8, 26] and immune cell–mediated fibrosis in other chronic models such as cardiac allograft vasculopathy [33].

Several limitations should be acknowledged. First, the use of a secondary intraperitoneal infection model bypasses early natural infection stages and may miss critical host–parasite interactions. Second, anti-ST2 treatment was initiated at an early stage of infection to evaluate its effects on parasite establishment and early lesion development; therefore, the therapeutic efficacy of ST2 blockade in established or late-stage disease remains to be determined. Third, although ST2 blockade reduced hepatic fibrosis and modulated immune responses, we cannot fully distinguish direct anti-fibrotic effects from improvements secondary to reduced parasite burden. Finally, systemic ST2 blockade affects multiple cell types, and the observed benefits may not reflect direct fibroblast-specific effects; cell type–specific ST2 manipulation will be required to dissect these mechanisms.

## Conclusions

Our study identifies the IL-33/ST2 axis as a conserved pathway involved in AE pathophysiology. Anti-ST2 treatment was associated with improved disease outcomes by restricting parasite growth and modulating the fibro-inflammatory immune environment. These findings provide a rationale for further investigation of ST2-targeted approaches as adjuncts to conventional antiparasitic strategies.

## Supplementary Information

**Additional file 1**. **Figure S1**: Additional representative immunohistochemistry (IHC) images of liver tissues.

**Additional file 2**. **Figures S2–S4**. Representative gross images from the experimental mouse model: **Figure S2**, metacestodes; **Figure S3**, liver lesions; **Figure S4**, spleens from control, AE-infected, and anti-ST2-treated mice.

## Data Availability

Data supporting the main conclusions of this study are included in the manuscript.

## Abbreviations

AE: Alveolar echinococcosis
*E. multilocularis*: *Echinococcus multilocularis*
ST2: Suppression of tumorigenicity 2
IL-33: Interleukin-33
sST2: Soluble ST2
IF: Immunofluorescence
IHC: Immunohistochemistry
HE: Haematoxylin and eosin staining
TEM: Transmission electron microscopy
M2: M2 macrophages
Th1/Th17: T helper 1/T helper 17 cells
B cells: B lymphocytes
CD4⁺ T cells: CD4-positive T lymphocytes
NK cells: Natural killer cells
HSCs: Hepatic stellate cells
TIMP-1: Tissue inhibitor of metalloproteinases 1
MMP-9: Matrix metalloproteinase 9
α-SMA: α-Smooth muscle actin
IFN-γ: Interferon gamma
TNF-α: Tumour necrosis factor alpha

## References

[1] Aydin F, Yalcin A, Karaman A, Sade R, Ozturk G, Alper F. Diagnostic and management perspectives in alveolar echinococcosis: review of literature. Eurasian J Med. 2022;54:10–5. 36655439 10.5152/eurasianjmed.2022.22308 PMC11163348

[2] Pavlidis ET, Galanis IN, Pavlidis TE. Current considerations for the management of liver echinococcosis. World J Gastroenterol. 2025;31:103973. 40093668 10.3748/wjg.v31.i10.103973 PMC11886533

[3] Zhang Z, Wang J, Li H, Niu Q, Tao Y, Zhao X, et al. The role of the interleukin family in liver fibrosis. Front Immunol. 2025;16:1497095. 39995661 10.3389/fimmu.2025.1497095 PMC11847652

[4] Kotsiou OS, Gourgoulianis KI, Zarogiannis SG. IL-33/ST2 axis in organ fibrosis. Front Immunol. 2018;9:2432. 30405626 10.3389/fimmu.2018.02432 PMC6207585

[5] Millar NL, O’Donnell C, McInnes IB, Brint E. Wounds that heal and wounds that don’t—The role of the IL-33/ST2 pathway in tissue repair and tumorigenesis. Semin Cell Dev Biol. 2017;61:41–50. 27521518 10.1016/j.semcdb.2016.08.007

[6] Zhou Y, Xu Z, Liu Z. Role of IL-33-ST2 pathway in regulating inflammation: current evidence and future perspectives. J Transl Med. 2023;21:902. 38082335 10.1186/s12967-023-04782-4 PMC10714644

[7] Pusceddu I, Dieplinger B, Mueller T. ST2 and the ST2/IL-33 signalling pathway-biochemistry and pathophysiology in animal models and humans. Clin Chim Acta. 2019;495:493–500. 31136737 10.1016/j.cca.2019.05.023

[8] Sheng F, Li M, Yu JM, Yang SY, Zou L, Yang GJ, et al. IL-33/ST2 axis in diverse diseases: regulatory mechanisms and therapeutic potential. Front Immunol. 2025;16:1533335. 39925809 10.3389/fimmu.2025.1533335 PMC11802536

[9] Pascual-Reguant A, Bayat Sarmadi J, Baumann C, Noster R, Cirera-Salinas D, Curato C, et al. T(H)17 cells express ST2 and are controlled by the alarmin IL-33 in the small intestine. Mucosal Immunol. 2017;10:1431–42. 28198366 10.1038/mi.2017.5

[10] Maggi L, Camelo GMA, Rocha IC, Pereira Alves W, Moreira JMP, Almeida Pereira T, et al. Role of the IL-33/ST2 activation pathway in the development of the hepatic fibrosis induced by Schistosoma mansoni granulomas in mice. Int J Mol Sci. 2023;24:10237. 10.3390/ijms241210237. 37373379 10.3390/ijms241210237 PMC10299179

[11] Autier B, Manuel C, Lundstroem-Stadelmann B, Girard JP, Gottstein B, Gangneux JP, et al. Endogenous IL-33 accelerates metacestode growth during late-stage alveolar echinococcosis. Microbiol Spectr. 2023;11:e0423922. 36786637 10.1128/spectrum.04239-22 PMC10101030

[12] Wu X, Ming B, Wu T, Gao R, Hu P, Tang J, et al. IL-33/ST2 axis contributes to the dermal fibrosis of systemic sclerosis via promoting fibroblasts activation. J Dermatol Sci. 2022;107:95–104. 35940987 10.1016/j.jdermsci.2022.07.009

[13] Song HK, Hwang DY. Use of C57BL/6N mice on the variety of immunological researches. Lab Anim Res. 2017;33:119–23. 28747977 10.5625/lar.2017.33.2.119 PMC5527137

[14] Zhu Y, Li M, Li Z, Song J, Zhao W. Study on the mechanism of miRNAs on liver injury in the condition of *Protoscocephalus alveolarus* transhepatic portal vein infection. Immun Inflamm Dis. 2024;12:e1236. 38652009 10.1002/iid3.1236 PMC11037255

[15] Abulizi A, Shao Y, Aji T, Li Z, Zhang C, Aini A, et al. *Echinococcus multilocularis* inoculation induces NK cell functional decrease through high expression of NKG2A in C57BL/6 mice. BMC Infect Dis. 2019;19:792. 31500589 10.1186/s12879-019-4417-1 PMC6734356

[16] Choi JY, Kim HR, Kim KS, Jung YS, Cho JY, Hwang DY, et al. Comparative study of the immunological characteristics of three different C57BL/6N mouse substrains. Lab Anim Res. 2017;33:124–31. 28747978 10.5625/lar.2017.33.2.124 PMC5527138

[17] Chuang CC, Chen CW, Huang YT, Du WY. Anti-ST2 monoclonal antibody inhibits eosinophil infiltration in *Angiostrongylus cantonensis*-infected mice. J Microbiol Immunol Infect. 2016;49:91–6. 24657070 10.1016/j.jmii.2014.01.009

[18] Cardiff RD, Miller CH, Munn RJ. Manual hematoxylin and eosin staining of mouse tissue sections. Cold Spring Harb Protoc. 2014;2014:655–8. 24890205 10.1101/pdb.prot073411

[19] Magaki S, Hojat SA, Wei B, So A, Yong WH. An introduction to the performance of immunohistochemistry. Methods Mol Biol. 2019;1897:289–98. 30539453 10.1007/978-1-4939-8935-5_25 PMC6749998

[20] Zaqout S, Becker LL, Kaindl AM. Immunofluorescence staining of paraffin sections step by step. Front Neuroanat. 2020;14:582218. 33240048 10.3389/fnana.2020.582218 PMC7680859

[21] Wu XL, Zou XY, Zhang M, Hu HQ, Wei XL, Jin ML, et al. Osteocalcin prevents insulin resistance, hepatic inflammation, and activates autophagy associated with high-fat diet-induced fatty liver hemorrhagic syndrome in aged laying hens. Poult Sci. 2021;100:73–83. 33357709 10.1016/j.psj.2020.10.022 PMC7772703

[22] Anderson NM, Simon MC. The tumor microenvironment. Curr Biol. 2020;30:R921–5. 32810447 10.1016/j.cub.2020.06.081 PMC8194051

[23] Aydin Y, Eren S, Ogul H. Pulmonary diffuse calcified multiple nodules in alveolar echinococcosis. Indian J Thorac Cardiovasc Surg. 2024;40:386–7. 38681702 10.1007/s12055-024-01685-x PMC11045688

[24] Wu S, Jiao J, Wang N, He N, Wu Y, Jiang H, et al. Tregs ST2 deficiency enhances the abscopal anti-tumor response induced by microwave ablation. Int Immunopharmacol. 2024;143:113330. 39423663 10.1016/j.intimp.2024.113330

[25] Nabekura T, Girard JP, Lanier LL. IL-33 receptor ST2 amplifies the expansion of NK cells and enhances host defense during mouse cytomegalovirus infection. J Immunol. 2015;194:5948–52. 25926677 10.4049/jimmunol.1500424 PMC4458425

[26] Di Carmine S, Scott MM, McLean MH, McSorley HJ. The role of interleukin-33 in organ fibrosis. Discov Immunol. 2022;1:kyac006. 38566909 10.1093/discim/kyac006 PMC10917208

[27] Yang YE, Hu MH, Zeng YC, Tseng YL, Chen YY, Su WC, et al. IL-33/NF-κB/ST2L/Rab37 positive-feedback loop promotes M2 macrophage to limit chemotherapeutic efficacy in lung cancer. Cell Death Dis. 2024;15:356. 38778059 10.1038/s41419-024-06746-y PMC11111460

[28] Wu Y, Shi W, Wang H, Yue J, Mao Y, Zhou W, et al. Anti-ST2 nanoparticle alleviates lung inflammation by targeting ILC2s-CD4(+)T response. Int J Nanomed. 2020;15:9745–58. 10.2147/IJN.S268282 PMC7721292 33299314

[29] Valeff NJ, Ventimiglia MS, Quadrana F, Jensen F. ST2-expressing B1 B cells acquire an anti-inflammatory capacity during pregnancy in mice. J Immunol. 2020;204:235.14-.14.

[30] Yin C, Liu B, Li Y, Li X, Wang J, Chen R, et al. IL-33/ST2 induces neutrophil-dependent reactive oxygen species production and mediates gout pain. Theranostics. 2020;10:12189–203. 33204337 10.7150/thno.48028 PMC7667675

[31] Lamberet A, Rostan O, Dion S, Jan A, Guegan H, Manuel C, et al. IL-33/ST2 axis is involved in disease progression in the spleen during Leishmania donovani infection. Parasit Vectors. 2020;13:320. 32571430 10.1186/s13071-020-04190-3 PMC7310124

[32] Larsen KM, Minaya MK, Vaish V, Peña MMO. The role of IL-33/ST2 pathway in tumorigenesis. Int J Mol Sci. 2018;19:2676. 10.3390/ijms19092676. 30205617 10.3390/ijms19092676 PMC6164146

[33] Zhang Z, Zhang N, Shi J, Dai C, Wu S, Jiao M, et al. Allograft or recipient ST2 deficiency oppositely affected cardiac allograft vasculopathy via differentially altering immune cells infiltration. Front Immunol. 2021;12:657803. 33815420 10.3389/fimmu.2021.657803 PMC8012811

