# Supplementary material for: ST2 blockade modulates IL-33–driven immune responses and is associated with reduced hepatic fibrosis in alveolar echinococcosis
Source: Parasit Vectors. 2026 Jun 16;19:361. doi: 10.1186/s13071-026-07462-6 (PMC13505141; doi:10.1186/s13071-026-07462-6)
Supplement: Supplementary file 1 [file 13071_2026_7462_MOESM1_ESM.docx]

**Fig. 2S: The IL-33/ST2 pathway is activated in human AE and attenuated by anti-ST2 therapy in mice.**

（I）COLI Human

|  |  |
| --- | --- |
| **Control（IHC x400）** | **infected（IHC x400）** |
|  |  |
| **Control（IHC x100）** | **infected（IHC x100）** |

（II）IL-6 Human

|  |  |
| --- | --- |
| **Control（IHC x400）** | **Infected（IHC x400）** |
|  |  |
| **Control（IHC x100）** | **Infected（IHC x400）** |

（III）IL-33 Human

|  |  |
| --- | --- |
| **Control（IHC x400）** | **Infected（IHC x400）** |
|  |  |
| **Control（IHC x100）** | **Infected（IHC x100）** |

### （IV）ST2 Human

|  |  |
| --- | --- |
| **Control（IHC x400）** | **Infected（IHC x400）** |
|  |  |
| **Control（IHC x100）** | **Infected（IHC x100）** |

（V）TIMP1 Human

|  |  |
| --- | --- |
| **Control（IHC x400）** | **Infected（IHC x400）** |
|  |  |
| **Control（IHC x400）** | **Infected（IHC x400）** |

(VI）α-SMA Human

|  |  |
| --- | --- |
| **Control（IHC x400）** | **Infected（IHC x400）** |
|  |  |
| **Control（IHC x100）** | **Infected（IHC x100）** |

（VII）MMP9 Human

|  |  |
| --- | --- |
| **Control（IHC x400）** | **Infected（IHC x400）** |

（I）COLI Mice

|  |  |
| --- | --- |
| **1 Treated 400X** | **2 Infected 400X** |
|  |  |
| **1 Treated 100X** | **2 Infected 100X** |
|  |  |
| **3 Control 400X）** | **3 Control 100X** |

（II）TIMP1 Mice

|  |  |
| --- | --- |
| **1 A Treated 400X** | **2 Infected 400X** |
|  |  |
| **1 Treated 100X）** | **2 Infected 100X** |
|  |  |
| **3 Control 400X）** | **3 Control 100X）** |

（III）α-SMA Mice

|  |  |
| --- | --- |
| **Treated 400X** | **Infected 400X** |
|  |  |
| **Treated 100X** | **Infected 100X** |
|  |  |
| **Control 400X** | **Control 100X** |

(IV) IL-6 Mice

|  |  |
| --- | --- |
| **Treated 400X** | **Infected 400X** |
|  |  |
| **Control 400X** | **Control 100X** |

(V)IL-33 Mice

|  |  |
| --- | --- |
| **Treated 400X** | **Infected 400X** |
|  |  |
| **Control 400X** | **Control 100X** |

(VI) ST2 Mice

|  |  |
| --- | --- |
| **Treated 400X** | **Infected 400X** |
|  |  |
| **Control 400X** |  |

(VII) MMP9 Mice

|  |  |
| --- | --- |
| **Treated 400X** | **Infected 400X** |
|  |  |
| **Control 400X** |  |

**(A)** Representative immunohistochemistry (IHC) images of ST2, IL-33, α-SMA, Collagen I, IL-6, TIMP1, and MMP9 in liver sections from human control, human AE, mouse control, mouse infected (AE model), and mouse treated (infected + anti-ST2) groups. Scale bar: 100 µm.

**Human Control:** Liver tissue from healthy human control subjects.

**Human AE:** Liver tissue from human patients with Alveolar Echinococcosis.

**Control Mouse:** Liver tissue from control mice.

**AE Mice:** Liver tissue from mice with experimentally induced Alveolar Echinococcosis.

**AE mice + Anti ST2 treated:** Liver tissue from AE mice treated with anti-ST2 neutralizing antibody.

**(B) Quantitative analysis of IHC staining intensity. Data are presented as mean ± SD. **Asterisks denote statistical significance (*p < 0.05, **p < 0.01, ***p < 0.001, ****p < 0.0001) for the indicated comparisons (one-way ANOVA with Tukey's post hoc test).**
