# Supplementary material for: ST2 blockade modulates IL-33–driven immune responses and is associated with reduced hepatic fibrosis in alveolar echinococcosis
Source: Parasit Vectors. 2026 Jun 16;19:361. doi: 10.1186/s13071-026-07462-6 (PMC13505141; doi:10.1186/s13071-026-07462-6)
Supplement: Supplementary file 2 [file 13071_2026_7462_MOESM2_ESM.docx]

**Fig. 3S1. Morphological and quantitative assessment of parasitic burden at 125 days post-infection.**

(A) **Metacestode Weight**: The metacestode weight in the anti-ST2-treated group was significantly reduced compared to the AE-infected group (p < 0.05), suggesting that the treatment effectively reduces parasitic burden.

(B) **Representative Abdominal Images**: Abdominal images depicting the metacestode masses in both AE-infected and anti-ST2-treated mice. The anti-ST2-treated group showed smaller metacestode masses, reflecting a reduction in parasitic load.

**Fig. 3S2.** **Morphological and quantitative assessment of parasitic burden on the liver at 125 days post-infection.**

**(A) Representative Liver Images:**
Liver images illustrating reduced lesion burden in anti-ST2-treated mice, where the lesions appeared smaller and less extensive compared to the AE-infected group.

**(B) Liver Lesion Size:**
Liver lesion size was significantly smaller in the anti-ST2-treated group (p < 0.01), with some mice showing no detectable lesions, indicating a strong therapeutic effect of anti-ST2 treatment.

**Fig. 3S3.** **Morphological and quantitative assessment of spleen size and shape at 125 days post-infection.**

**(A) Spleen Size:**
Representative images showing the spleen size in AE-infected and anti-ST2-treated mice at 125 days post-infection. The spleen of anti-ST2-treated mice appeared smaller compared to AE-infected controls.

**(B) Quantitative Analysis:**
Quantitative analysis of spleen size revealed a significant reduction in spleen weight in the anti-ST2-treated group (p < 0.01 vs. control; ns vs. AE-infected), indicating a partial reversal of the immune response and reduced splenomegaly.
